# Metabolic Costs of Emerging Contaminants: Cellular Energy Allocation in Zebrafish Embryos

**DOI:** 10.3390/jox15040099

**Published:** 2025-06-29

**Authors:** Bárbara S. Diogo, Daniela Rebelo, Sara C. Antunes, Sara Rodrigues

**Affiliations:** 1 Abel Salazar Biomedical Sciences Institute (ICBAS), University of Porto, Rua de Jorge Viterbo Ferreira, 228, 4050-313 Porto, Portugal; barbara.diogo@fc.up.pt (B.S.D.); up202210683@edu.icbas.up.pt (D.R.); 2 Centre Interdisciplinary of Marine and Environmental Research, Laboratory Associated (CIIMAR/CIMAR, LA), University of Porto, Terminal de Cruzeiros do Porto de Leixões, 4450-208 Matosinhos, Portugal; scantunes@fc.up.pt; 3 Department of Biology, Faculty of Sciences, University of Porto (FCUP), Rua do Campo Alegre S/N, 4169-007 Porto, Portugal

**Keywords:** antibiotics, aromatic amines, *Danio rerio*, available energy, energy consumed

## Abstract

The use of cellular energy allocation (CEA) as a physiological energetic biomarker is useful for detecting the sublethal effects of environmental contaminants. The CEA assesses the health and energy status of organisms, serving as a reliable indicator for monitoring the health of aquatic ecosystems. This study aimed to evaluate the impact of emerging contaminants already listed as a priority for monitoring in freshwater ecosystems, namely sulfamethoxazole (0.156–2.50 mg/L), trimethoprim (25.0–400 mg/L), 4-chloroaniline (5.21–20.0 mg/L), and 3,4-dichloroaniline (0.38–4.00 mg/L), on the CEA of *D. rerio* embryos. A standard fish embryo toxicity test was conducted, and an adaptation of the allometric scaling approach was developed through the relationship between the size and the fresh weight of the embryos. All the compounds affected the fractions of the energy reserves (total carbohydrate, lipid, and protein contents) differently, with carbohydrates being the predominant energy fraction and the most responsive indicator. Although the energy consumed showed no significant changes, the CEA was notably altered after exposure to all the contaminants, indicating a direct connection to shifts in the available energy. The CEA alterations may indicate a reallocation of energy toward detoxification, combating the stress of contaminant exposure. Energy allocation biomarkers provide a comprehensive assessment of an organism’s physiological state, which is essential for evaluating emerging contaminants’ impacts, safeguarding aquatic ecosystems, and shaping effective environmental policies.

## 1. Introduction

The presence of emerging contaminants in different environmental matrices (e.g., sediment, soil, and water) worldwide has become a major concern for society, public health authorities, and the scientific community [1]. The continuous input of these contaminants into ecosystems can impact non-target organisms (e.g., microcrustaceans and fish) with important ecological functions and trigger different adverse effects (e.g., mortality and reproductive impairments) [2], with potentially harmful consequences for the balance of ecosystems. These contaminants can interact with specific biological systems, such as enzymes or receptors, even at low concentrations (µg/L or ng/L), triggering different metabolic/physiological responses and compromising the normal functioning of organisms and animal welfare (e.g., Rebelo et al., [3]; Diogo et al., [4]). In more severe cases, disturbances at higher levels of biological organization and alterations in the ecosystem dynamics can occur (e.g., community structure, ecosystem services) [4,5].

In recent years, due to their widespread use, as well as their persistence in the environment, several compounds have been included, or selected as suitable candidates for inclusion, on the Watch List (WL) of priority substances under the Water Framework Directive (WFD) to be monitored in inland surface waters across the European Union [6,7]. This list serves as an early warning system for emerging contaminants, addressing knowledge gaps about them and collecting high-quality data to assess the risk they may pose to ecosystems [6,7,8]. Sulfamethoxazole (SMX) and trimethoprim (TRIM) are antibiotics already considered priority substances in the third WL and carried over to the fourth WL due to their widespread use (e.g., human and veterinary medicine, aquaculture) and potential toxicity to aquatic organisms [6,7]. On the other hand, 4-chloroaniline (4-CA) and 3,4-dichloroaniline (3,4-DCA), two aromatic amines used in the production of dyes, cosmetics, pharmaceuticals, laboratory chemicals, and pesticides, are still considered suitable candidates for the fourth WL, since the available toxicity data is scarce [7]. These four compounds have already been detected in different aquatic matrices worldwide (e.g., fresh, marine, and groundwater), at levels ranging from ng/L to μg/L (surface water: 150 µg/L of SMX; 30 µg/L of TRIM; 67 µg/L of 4-CA; 20 µg/L of 3,4-DCA), and are considered to be persistent in the environment [3,9,10]. However, the potential ecotoxicological effects they can have on aquatic ecosystems are still unknown. Although some studies have already reported the ecotoxicological effects caused in different aquatic organisms, the available data about their effects at sub-individual levels (e.g., antioxidant defense enzymes, energetic reserves, neurotransmission) is incomplete [3,8]. Understanding the sub-individual effects of pollutants enables early toxicity detection, clarifies mechanisms of action, and allows for a more sensitive assessment of ecological and human risks, guiding effective environmental policies.

Over the past few decades, different biomarkers have been used as early warning tools in environmental quality assessment to investigate the sublethal effects of different environmental contaminants [2,11,12]. Moreover, several authors highlight the importance of studying biomarkers related to energetic metabolism [13,14,15,16] since it plays a crucial role in organisms’ physiological and behavioral functions. The energetic metabolism provides information beyond responses to biochemical, cellular, and consequently, growth and reproductive functions, exhibiting long-term effects on different levels of biological organization (e.g., organs, organisms, populations) [13,14]. Different studies have demonstrated the association between biochemical alterations related to energy pathways and the impairment of other physiological or behavioral functions in non-target organisms exposed to different stressors [17,18,19]. In this sense, the cellular energy allocation (CEA) is an approach used to assess the health and energetic status of organisms exposed to environmental stressors, such as emerging contaminants [17]. The difference between the available energy reserves (measured by the total carbohydrate, lipids, and protein contents) and the energy consumed (measured by the electron transport system activity) for different cellular functions provides an integrated view of the body’s physiological condition [13]. Alterations in the CEA, depending on their magnitude, can be correlated with alterations in individual parameters of the organisms’ life cycle (e.g., growth and reproduction), which can compromise the energy allocation for other physiological processes, and consequently, affect the population structure and dynamics [5,16,20]. CEA changes can also provide insights into sublethal impacts and long-term adaptive capacity.

Aderemi et al. [21] demonstrated the relevance of evaluating the potential impact of substances identified as high risk for the aquatic environment through CEA assessment in aquatic organisms, namely the microalga *Raphidocelis subcapitata*. These authors studied the effect of three priority substances included in the first WL(ciprofloxacin, erythromycin, and clarithromycin [22]) and reported the CEA as a reliable indicator of the physiological status of the organisms that could be useful in monitoring aquatic ecosystem health [21]. This biomarker has already been applied in several model organisms (e.g., *Daphnia magna, Chironomus riparius*) [5,23,24]; however, few studies have used this approach in *Danio rerio* embryos (a model organism widely used in ecotoxicological studies). Thus, the present study aims to evaluate the effect of emerging contaminants already considered priority substances (SMX and TRIM) and suitable candidates (4-CA and 3,4-DCA) for inclusion in the current WL in the cellular energy allocation of *D. rerio* embryos. Studying the effects of emerging contaminants on aquatic organisms is essential for protecting biodiversity and ensuring ecosystem health. Focusing on the CEA provides valuable insights into how these contaminants affect the organism’s health. Moreover, these biomarkers serve as an early warning system, indicating changes before individual-level effects occur, thus helping to protect ecosystem quality. By understanding these impacts, we can inform effective environmental policies prioritizing aquatic systems’ health and the organisms within them.

## 2. Materials and Methods

### 2.1. Chemicals and Test Solutions

Sulfamethoxazole (SMX), trimethoprim (TRIM), 4-chloroaniline (4-CA), and 3,4-dichloroaniline (3,4-DCA) were acquired from Sigma Aldrich (Merck - Portugal). All the compounds’ properties, stock solutions, and concentrations tested are listed in Table 1. The stock solutions were prepared by dilution of each compound in sterile dechlorinated water retrieved from the recirculating system (the same as laboratory broodstock), and the concentrations tested were selected based on the relevant literature data (Table 1) [9,25,26,27,28], including the reported median lethal concentration (LC_50_), LC_30_, no observed effect concentration (NOEC), and other effect thresholds. This selection allowed us to cover a biologically meaningful range, from concentrations expected to produce no observable effects to those associated with sublethal and lethal outcomes.

### 2.2. Danio rerio Embryos

*Danio rerio* is a freshwater fish recognized as a model organism, which is used in ecotoxicological studies [29]. Zebrafish embryos are often used for evaluating morphological and behavioral changes, as well as for biochemical analyses [29]. As a result, their application in environmental risk assessments is growing, and they are increasingly recommended for testing the toxicity of a wide range of chemical pollutants [29]. The embryos used in the experiment were acquired from a laboratory broodstock (wildtype AB) of the zebrafish facility at the CIIMAR—Interdisciplinary Centre of Marine and Environmental Research (Matosinhos, Portugal). *Danio rerio* adults were kept in aquariums in a recirculating water system, in laboratory-certified facilities at the CIIMAR (Matosinhos, Portugal), under controlled conditions of temperature of 26 ± 1 °C, pH = 7.5 ± 0.5, conductivity of 200–300 μS/cm, dissolved oxygen (O_2_) ≥ 80% of saturation, photoperiod of 16h^L^: 8h^D^, ammonium (NH_4_) and nitrites (NO_2_) < 0.001 mg/L. The individuals were fed *ad libitum* once daily with dry commercial fish food (Tetra Goldfish). Glass marbles were placed at the bottom of the reproduction aquarium to protect the eggs from predation by the progeny. After removing the marbles, the eggs were collected and rinsed with water from the recirculating system. Viable eggs (without irregularities) were identified under a stereomicroscope (Carl Zeiss Stemi DV4, Germany) as quickly as feasible (<90 min post-fertilization; <16 cell-stage), and the viable eggs (identified by the development of a blastula) were used to conduct the bioassays.

### 2.3. Allometric Estimation of Fresh Weight in Danio rerio Embryos

**Figure 1:**
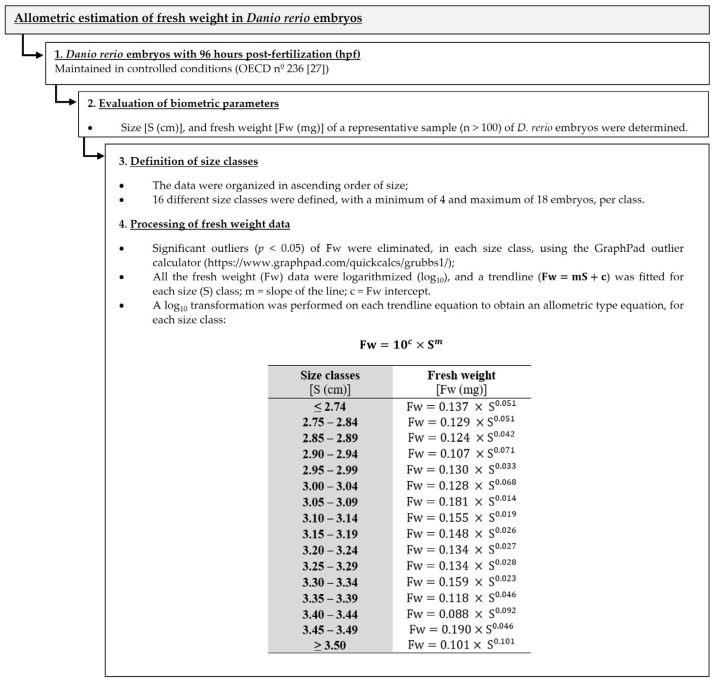
Sequential flowchart illustrating the steps taken to obtain the allometric estimation of the fresh weight in *Danio rerio* embryos (96 h post-fertilization—hpf).

Several authors have already used allometric scaling to investigate the relationship between two variables in different model organisms (e.g., body mass and metabolic rate; body size and organs; exopodite length and total body length) [30,31,32]. An adaptation of the allometric scaling approach [30,31] was developed in this study through the relationship between the size and the fresh weight of *D. rerio* embryos (Figure 1). Weight is a key parameter for the analysis of biochemical biomarkers (e.g., energy metabolism biomarkers), as it allows the standardization of the results, facilitating comparison between samples and ensuring data accuracy. Given the extreme fragility of zebrafish embryos and the impracticality of handling, weighing, and preparing them for multiple analyses within the limited time frame of a short-term exposure experiment, especially with the use of highly sensitive ultra- and micro-analytical balances that require significant time for individual measurements, the allometric estimation of the *D. rerio* embryos’ fresh weight presented here overcomes these challenges by allowing timely and appropriate weight estimation, immediately after exposure, without compromising subsequent biochemical analyses, making it essential for future research (Figure 1).

### 2.4. Bioassay Conditions

Zebrafish embryo acute toxicity assays were conducted according to the standard Fish Embryo Acute Toxicity (FET) test [27], with some modifications (e.g., eggs were transferred to other 24-well plates with renewal medium after 48 h). To perform the bioassays, 24-well microplates were used, with 20 replicates per concentration tested, and one embryo per replicate. Each well contained 2 mL of each treatment (previously prepared; Table 1) or sterile dechlorinated water retrieved from the recirculating system (control group). The microplates were maintained under controlled conditions of temperature (26 ± 1 °C) and photoperiod (16 h^L^:8 h^D^) for 96 hpf [27]. The test medium was entirely renewed after 48 h. During the assay (after 24, 48, 72, and 96 h of exposure), all the coagulated eggs or dead embryos were counted and removed. After 96 h of exposure, three pools/replicates (with 2 organisms each) were randomly collected from each concentration. The organisms were measured using a binocular stereoscope (Leica MZ 75 with an attached camera Leica DFC 290, Germany) and then stored in Eppendorf microtubes at −80 °C for subsequent assessment of the cellular energy allocation (CEA) effects. The fresh weight of each pool/replicate (i.e., sum of the weights of organisms based on their respective sizes) was obtained considering the allometric estimation of the fresh weight in *Danio rerio* embryos (Figure 1) and used to express the different energetic pathways.

### 2.5. Cellular Energy Allocation (CEA)

Samples were defrosted on ice and homogenized in 1 mL of ice-cold phosphate buffer (50 mM, pH 7.0). The homogenate was divided into three aliquots for the determination of the (i) carbohydrate content (300 µL), (ii) lipid content (300 µL), and (iii) protein content and the electron transport system (ETS) activity (300 µL) analysis.

#### 2.5.1. Available Energy Reserves (Ea)

To quantify the carbohydrate content, 100 µL of 15% trichloroacetic acid was added to the aliquots and held at −20 °C for 10 min. After that, the samples were centrifuged at 10,000 rpm for 5 min at 4 °C. To 250 µL of the supernatant fraction were added 250 µL of 5% (*v*/*v*) phenol and 1 mL of H_2_SO_4_ (95–97%) (adapted from De Coen and Janseen [13]). After 30 min of incubation at 20 °C, the absorbance was measured at 492 nm, using glycogen as a standard. The lipid extraction procedure was performed through the biphasic solvent system consisting of chloroform/methanol/water, after centrifuging the aliquots at 10,000 rpm for 5 min [33,34]. For the protein content quantification was used 40 µL from the protein and ETS activity aliquot, after adding 150 µL of buffer (0.3 M Tris; 15% (*w*/*v*) Poly Vinyl Pyrrolidone; 8 mM MgSO_4_; 0.6% (*v*/*v*) Triton X-100) and centrifuging at 3500 rpm for 10 min at 4 °C. The protein quantification was performed at 595 nm using the Bradford method, adapted to a microplate with bovine γ-globulin as the standard [35].

All these energetic reserves are expressed by mg of fresh weight, regarding the allometric estimation of the fresh weight in *Danio rerio* embryos (Figure 1). The available energy reserves (Ea) were estimated by Equation (1):

(1) Ea=carbohydrates+lipids+protein contents

where the total carbohydrate, lipid, and protein contents were transformed into energetic equivalents by the enthalpy of combustion (24,000 mJ/mg protein, 39,500 mJ/mg lipids, and 17,500 mJ/mg glycogen), according to De Coen and Janseen [13].

#### 2.5.2. Energy Consumed (Ec)

The electron transport system (ETS) activity was quantified according to De Coen and Janseen [13], with some modifications as described below. The remaining ETS aliquots were centrifuged (3500 rpm, 10 min, 4 °C) after the addition of 150 µL of buffer [0.3 M Tris; 15% (*w*/*v*) Poly Vinyl Pyrrolidone; 8 mM MgSO_4_; 0.6% (*v*/*v*) Triton X-100]. After this, 50 µL of the supernatant fraction was added to 150 µL of buffered substrate solution [0.13 M Tris HCl, 0.3% (*w*/*v*) Triton X-100, pH 8.5] and 50 µL of NAD(P)H solution (1.8 mM NADH; 280 μM NADPH). The reaction was started by adding 100 µL of *p*-Iodonitrotetrazolium (INT, 8 mM), and the absorbance was measured at 490 nm for 10 min.

The cellular respiration rate (determined using the ETS methodology) was based on a theoretical stoichiometric relationship (2 mmol INT–formazan formed for 1 mmol oxygen consumed) described by De Coen and Janseen [13]. The consumed oxygen was calculated according to Lambert–Beer (Equation (2)):

A = ε × l × c(2)

where A = absorbance; ε for INT formazan = 15,900/M.cm; l = 0.9 cm; and c = oxygen consumed in M. The values were transformed into energetic equivalents using the oxyenthalpic equivalent for the average carbohydrate, lipid, and protein mixture (480 kJ/mol O_2_; [13]). The Ec (measured by the ETS activity) was expressed in mJ/mg fresh weight/min (Figure 1).

#### 2.5.3. Cellular Energy Allocation (CEA) Calculation

The CEA considers the relationship between the total carbohydrate, lipid, and protein contents and the ETS activity, being calculated using Equation (3):

(3) $$CEA=\frac{Ea\;(carbohydrates+lipids+protein\;contents)}{Ec\;(ETS\;activity)}$$

where Ea = available energy reserves (mJ/mg fresh weight); Ec = energy consumed (mJ/mg fresh weight/min); and the final CEA value is stated as hours^−1^.

### 2.6. Statistical Analysis

All the results obtained in the biomarker determinations were tested for normality and homogeneity by the Shapiro–Wilk and Levene tests, respectively. After the confirmation of these assumptions, a one-way ANOVA was performed for all the biomarker results, and when significance was detected, a Dunnett’s test was performed to discriminate the differences between the compound concentrations and the control group. SPSS Statistics v29 was used for all the statistical analyses, using a significance level of α = 0.05.

## 3. Results

**Figure 2:**
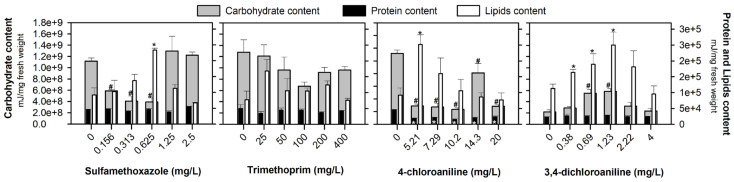
Available energy fractions (total carbohydrate, lipid, and protein contents) (mean of 3 replicates ± SE) of *Danio rerio* embryos after 96 h of exposure to the antibiotics (sulfamethoxazole and trimethoprim), and aromatic amines (4-chloroaniline and 3,4-dichloroaniline). # Stands for significant differences in the carbohydrate content compared to the control treatment (Dunnett’s test, *p* < 0.05). The white asterisk (*) stands for significant differences in the protein content, and the black asterisk (*) stands for significant differences in the lipid content, compared to the control treatments (Dunnett’s test, *p* < 0.05).

Figure 2 shows the individual energy reserve fractions (total carbohydrate, lipid, and protein contents) of *D. rerio* embryos after 96 h of exposure to SMX, TRIM, 4-CA, and 3,4-DCA. Overall, carbohydrates had the highest fraction of the energy reserve, and a significant decrease after exposure to the lowest concentrations of SMX tested (F_[5, 17]_ = 11.866, *p* < 0.001) was observed. The same response was observed for all the 4-CA concentrations tested (F_[5, 17]_ = 26.975, *p* < 0.001), whilst a significant increase occurred after exposure to 0.69 and 1.23 mg/L of 3,4-DCA (F_[5, 17]_ = 10.058, *p* < 0.001). No significant alterations in the carbohydrate values were observed after TRIM exposure (F_[5, 17]_ = 1.775, *p* = 0.193). Regarding the lipid content (white bars in Figure 2), a significant increase was observed after exposure to 0.625 mg SMX/L (F_[5, 17]_ = 4.396, *p* = 0.017), 5.21 mg 4-CA/L (F_[5, 17]_ = 4.632, *p* = 0.014) and 1.23 mg 3,4-DCA/L (F_[5, 17]_ = 3.000; *p* = 0.055). After TRIM exposure, no significant alterations were found in the lipid content (F_[5, 17]_ = 1.808, *p* = 0.186; Figure 2). The protein content was the least expressive reserve energy, and no significant alterations were observed after exposure to SMX (F_[5, 17]_ = 5.603, *p* = 0.007), TRIM (F_[5, 17]_ = 0.716, *p* = 0.623) and 3,4-DCA (F_[5, 17]_ = 0.259, *p* = 0.927). However, a significant decrease was recorded after exposure to all the concentrations tested of 4-CA (F_[5, 17]_ = 23.249, *p* < 0.001; Figure 2).

**Figure 3:**
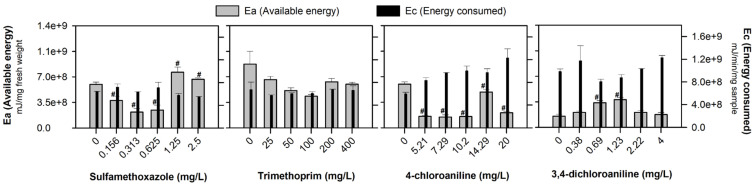
Available energy (Ea) and energy consumed (Ec) (mean of 3 replicates ± SE) in *Danio rerio* embryos after 96 h of exposure to the antibiotics (sulfamethoxazole and trimethoprim) and aromatic amines (4-chloroaniline and 3,4-dichloroaniline). # Stands for significant differences in the Ea compared to the control treatment (Dunnett’s test, *p* < 0.05).

**Figure 4:**
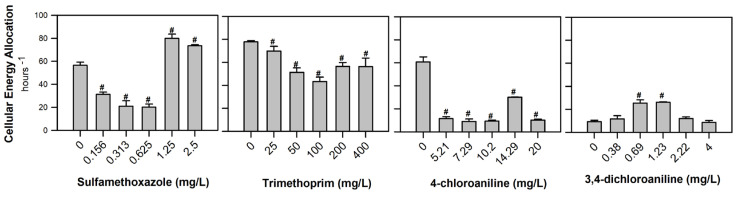
Cellular energy allocation (CEA) (mean of 3 replicates ± SE) in *Danio rerio* embryos after 96 h of exposure to the antibiotics (sulfamethoxazole and trimethoprim) and aromatic amines (4-chloroaniline and 3,4-dichloroaniline). # Stands for significant differences compared to the control treatment (Dunnett’s test, *p* < 0.05).

The results of the available energy (Ea), energy consumed (Ec), and cellular energy allocation (CEA) of *D. rerio* embryos after exposure to a range of concentrations of SMX, TRIM, 4-CA, and 3,4-DCA are presented in Figure 3 and Figure 4, respectively. The Ea values decreased significantly after exposure to the lowest SMX concentrations (F_[5, 17]_ = 11.863, *p* < 0.001) and all the 4-CA concentrations tested (F_[5, 17]_ = 26.9621; *p* < 0.001). The opposite occurred after exposure to 0.69 and 1.23 mg of 3,4-DCA/L (F_[5, 17]_ = 10.055; *p* < 0.001). No significant alterations in the Ea values were observed after TRIM exposure (F_[5, 17]_ = 1.775, *p* = 0.193). No significant changes were observed in the Ec after exposure to all the tested compounds: SMX (F_[5, 17]_ = 1.333, *p* = 0.315), TRIM (F_[5, 17]_ = 0.211, *p* = 0.951), 4-CA (F_[5, 17]_ = 1.407; *p* = 0.290) and 3,4-DCA (F_[5, 17]_ = 0.661; *p* = 0.660; Figure 3).

The CEA results revealed a significant decrease after exposure to all the concentrations of TRIM (F_[5, 17]_ = 8.357, *p* < 0.001) and 4-CA (F_[5, 17]_ = 88.562; *p* < 0.001). Exposure to SMX induced a dual response, with a significant decrease recorded in the lowest concentrations and an increase after 1.25 mg/L (F_[5, 17]_ = 75.126, *p* < 0.001). A significant increase in the CEA values was also observed after exposure to 0.69 and 1.23 mg of 3,4-DCA/L (F_[5, 17]_ = 18.424, *p* < 0.001; Figure 4).

## 4. Discussion

Biological processes in zebrafish embryos often show complex or non-linear responses, especially with multiple contaminants. In the present study, while a clear concentration–response trend was not observed, the statistically significant differences from the control suggest that the tested emerging contaminants had an effect, even if it was not directly dose-dependent. The differences noted in the control groups across the contaminants can be attributed to intraspecific variability among the zebrafish, differences in broodstocks (distinct groups of breeding fish), and the fact that the exposures were conducted at different times rather than simultaneously. All the bioassays were conducted under standardized and rigorously controlled laboratory conditions, ensuring the health and viability of the embryos used; therefore, the differences observed between the control groups across the contaminants cannot be attributed to the husbandry or culturing conditions. Different authors have noted that organisms often initiate compensatory adjustments (e.g., in energy metabolism) to maintain physiological homeostasis after exposure to environmental stressors, which, along with the potential threshold effects, could explain the observed patterns [13,19,33].

The emerging contaminants under study affected the energy reserve fractions (total carbohydrate, lipid, and protein contents) differently, and the most outstanding response was the depletion of the carbohydrate levels, except for 3,4-DCA (Figure 2). Carbohydrates are quick energy sources [36], and their depletion is already attributed to their critical role in maintaining the energy balance under stress conditions [37]. This type of response has been observed after exposure to different compounds (e.g., contaminants and effluents) and in different species (e.g., *Dreissena polymorpha, Danio rerio, Daphnia magna*) [14,38,39], which may be due to enhanced glycolytic activity and/or inhibition of gluconeogenesis [40]. Diogo et al. [8] reported that the same SMX concentrations (0.156 and 0.313 mg/L) induced oxidative stress in zebrafish embryos through an increase in the catalase and glutathione S-transferases activities. Increased metabolism and antioxidant defense activity may redirect glucose toward NADPH production, significantly lowering the carbohydrate levels [41]. This effect is likely a response to counteract oxidative stress [41]. In contrast to the observed tendency in SMX and 4-CA, an increase in the carbohydrate content at 0.69 and 1.23 mg of 3,4-DCA/L (Figure 2) can indicate a temporary adaptive shift concerning the stress. This shift suggests that glycogenesis may have converted glucose into glycogen to meet the detoxification demands, potentially inhibiting glycolysis and leading to carbohydrate accumulation [42].

Lipids are crucial for cell membranes, signal transmission, energy storage, and metabolic pathways, exhibiting high conservation across organisms [43]. In the present study, despite the significant increases observed in the zebrafish embryos after exposure to the tested compounds, these results occur occasionally (at a single concentration of SMX and 4-CA, and in the three lowest concentrations of 3,4-DCA; Figure 2). These alterations may reflect early metabolic adjustments, as organisms under acute stress can redirect energy usage, upregulate metabolism, or modulate lipid mobilization as part of their coping strategies. Studies have already reported that SMX promotes lipogenesis, reduces lipolysis, and increases the size of lipid droplets in *Drosophila melanogaster* larvae [44]. Park et al. [45] also reported that 3,4-DCA treatment led to a dose-dependent increase (≥1.62 mg/L) in hepatic lipid accumulation and upregulation of lipogenesis-related genes in zebrafish larvae after extended exposure, indicating that this response may persist under longer exposure periods. Other previous studies already reported that antibiotics (e.g., sulfamethazine and azithromycin) and aromatic amines (e.g., 3,4-DCA) can cause a disruption to lipids metabolism through different mechanisms in *D. rerio* embryos, either through increased oxidative stress, the redirection of energy from lipid storage to detoxification, or a change in gene expression related to lipid synthesis [44,45,46,47]. However, considering the short exposure duration in our study (96 h, without feeding), the observed lipid accumulation may represent a transient adaptive response to chemical stress rather than stable lipogenic reprogramming. This interpretation is supported by previous studies suggesting that shifts in energy reserves, including lipid fluctuations, often reflect temporary coping mechanisms under environmental stress rather than lasting physiological alterations [48]. Although variations in the protein levels of zebrafish have been reported after exposure to different compounds (e.g., Diogo et al., [33]), in the present study, the protein levels did not change after exposure to SMX, TRIM, and 3,4-DCA (Figure 2). These findings underscore the need for further investigation into the long-term effects of these contaminants on lipid homeostasis. This may be because proteins are typically the last energy source the body utilizes [33]. Proteins serve as energy sources, mobilized under severe stress or high energy demand [38,49], as potentially observed in this work, namely for 4-CA. The reduction in protein after 4-CA exposure (Figure 2) may stem from inhibited ribosomal function, reduced protein synthesis gene expression, or increased proteolytic activity [50,51]. Additionally, reactive oxygen species (ROS) formation due to 4-CA exposure can cause oxidative stress, leading to protein damage [52]. Increased oxidative stress (caused by an increase in ROS) can accelerate protein degradation and enhance the demand for protein synthesis to prevent damage [53]. Inhibition of detoxification enzymes may further exacerbate oxidative stress and compromise the organism’s ability to repair and synthesize essential proteins, leading to a decrease in the overall protein content [33]. The reduction in the protein content can impair several cellular functions, including the structural integrity, enzymatic activity, and metabolic regulation [8,53].

As a sum of the different energy reserve fractions, a decrease in the Ea generally indicates a high energy demand in response to adverse environmental conditions or chemical stresses [16,20]. The observed decrease after SMX and 4-CA exposure (Figure 3) may arise from increased metabolic activity or biological processes that require more energy (e.g., cellular repair and maintenance) [54]. Antibiotics and aromatic amines could induce cellular stress, increasing metabolic activity as the body attempts to repair damage, combat oxidative stress, and maintain vital functions, which can be time- and concentration-dependent [3,21,55]. Previous studies performed with the contaminants under study (at the same range of concentrations) showed that they can affect different pathways, such as oxidative stress, oxidative damage, and genotoxicity, which can influence energy expenditure [3,8]. However, activating these defense mechanisms requires additional energy, potentially altering the energy balance and overall physiological state of the organisms. The significant increase in the Ea after 3,4-DCA exposure (Figure 3) might indicate heightened metabolic activity to counteract toxic stress, potentially reallocating energy toward defense mechanisms against oxidative damage [56]. Consequently, the available energy fluctuations observed in the present study, specifically in the carbohydrates, can be caused by the reallocation of resources to prioritize detoxification processes [16,42]. According to Diogo et al. [8], the same SMX concentrations that decrease the carbohydrate content also stimulate the mobilization of antioxidant defense enzymes in zebrafish embryos, preventing lipid peroxidation.

The Ec (measured by the ETS activity) was not significantly affected by the emerging contaminants in the present study (Figure 3), under the conditions tested here, suggesting a balanced ATP demand and energy expenditure [49,57]. The ETS is a multi-enzyme complex situated in the inner mitochondrial membrane and serves as the final stage of cellular respiration [58]. Its activity, which encompasses ATP production and electron transfer, has been correlated with oxygen consumption across different groups of organisms [58]. The results obtained showed the ability of zebrafish embryos to maintain the Ec, indicating that under stressful conditions, the embryos tried to mitigate the contaminants’ impact by limiting their metabolic activity (e.g., embryos can maintain suitable ATP production and preserve essential cellular functions). Several authors have reported that to maintain physiological homeostasis after exposure to different environmental stressors, organisms initiate compensatory adjustments in energy metabolism (e.g., ATP production) [13,19,33]. Although the ETS activity may eventually reflect changes in the environment, its response to acute or short-term stressors is not immediate [59]. This slowness reflects the inherent stability of the ETS system and the time required for enzymatic adjustments and metabolic changes to be implemented [58].

Different factors (e.g., abiotic stressors, contaminants, illnesses) can affect an organism’s available and consumed energy, causing shifts in the cellular energy allocation [37,60]. The CEA decreased significantly after exposure to SMX (lowest concentrations), TRIM, and 4-CA (Figure 4) due to a reduction in the available energy [5,60]. On the contrary, the highest concentrations of SMX (1.25 and 2.5 mg/L) and 3,4-DCA exposure revealed an increase in the CEA (Figure 4), which is directly related to an increase in the available energy, specifically carbohydrates (Figure 2 and Figure 3). A decrease in the CEA suggests that organisms are under stress, reallocating energy toward detoxification processes at the expense of growth and reproduction. On the other hand, under favorable conditions, an increase in the CEA can represent a reallocation of energy for growth and reproduction, and an increase or replacement of the energy reserves. Aderemi et al. [21] and Alzahrani et al. [61] studied the energy metabolism effects of SMX and 3,4-DCA in the microalgae *Raphidocelis subcapitata* and the rotifer *Brachionus calyciflorus*, respectively. Both groups of authors found a significant reduction in the Ea after exposure to 0.24 mg of SMX/L and 0.4–0.8 mg of 3,4-DCA/L. Furthermore, a decrease in the Ec in *R. subcapitata* was observed after exposure to 0.24 mg of SMX/L, while higher concentrations (1.58, 2.96, and 8.30 mg/L) caused an increase. An increase in the Ec was also evident after exposure to 0.2–0.8 mg of 3,4-DCA/L in *B. calyciflorus*. These authors also identified that the CEA was significantly affected by concentrations of 2.96 and 8.3 mg of SMX/L and 0.4–0.8 mg of 3,4-DCA/L [21,60]. In addition to these biochemical alterations, organism-level effects have also been reported for the same contaminants. Diogo et al. [8] observed increasing morphological abnormalities (e.g., pericardial edema, body curvature, and enlarged swim bladder) in zebrafish embryos exposed to SMX and TRIM, with up to 85% abnormality at 2.5 mg of SMX/L and 50% at 400 mg of TRIM/L. Furthermore, the survival decreased to 85% and 95% after 48 h of exposure to SMX and TRIM, respectively. For 4-CA and 3,4-DCA, Rebelo et al. [62] reported >60% malformations (e.g., yolk sac oedema, hemagglutination, hypopigmentation) from the lowest 4-CA concentration (5.21 mg/L) and ≥2.22 mg/L of 3,4-DCA, along with increased mortality at 48 and 72 h. Notably, these studies used zebrafish embryos from the same origin and rearing conditions, and they also reported consistent responses in the control groups, further supporting the reliability and reproducibility of the data presented here. Together, the organism-level outcomes, alongside the observed energetic disruptions, underscore the relevance of linking sublethal biochemical markers like the CEA to higher-level biological effects. This connection provides valuable insight into how early cellular responses can foreshadow broader ecological consequences.

Smolders et al. [38] also showed that complex mixtures (e.g., effluents and environmental samples) could impair the total available energy of adult zebrafish after long periods of exposure (28 days). Abe et al. [16] investigated the effect of synthetic dyes on the energy metabolism parameters of *D. rerio* embryos, reporting a decrease in the Ec of the embryos. This effect compromised the energy balance and consequently the Ea for biological processes, including locomotor activity. Changes in the levels of available and consumed energy can impair physiological processes (e.g., growth and reproduction), reducing the fitness and survival of organisms [14,38]. This can result in lower reproductive output and increased mortality, leading to population decline and ultimately altering the community composition and ecosystem productivity [5]. The CEA is an important biomarker for the individual’s energy assessment, which can be influenced by environmental factors, nutritional status, and physiological conditions [13,14,38].

## 5. Conclusions

The fractions of the energy reserves and energy consumed were affected differently by the antibiotics (SMX and TRIM) and aromatic amines (4-CA and 3,4-DCA), revealing that the allocation of cellular energy in *D. rerio* embryos was influenced by these contaminants, corroborating the ecotoxicological effects reported at similar concentrations in the literature. Among the endpoints determined in the cellular energy allocation of *D. rerio* embryos, the carbohydrate content emerged as the predominant energy fraction and the most responsive indicator in compound exposures. Energy allocation biomarkers are a sensible endpoint to study priority substances and emerging contaminants as they demonstrate how contaminants disrupt the energy distribution within organisms, affecting critical processes (e.g., growth and reproduction). This helps to provide deeper insight into the long-term ecotoxicological effects. The energy mobilization induced by SMX, TRIM, and 4-CA may be directed toward managing stress and reducing the energy available for other vital physiological functions. Energy allocation biomarkers, such as the CEA, offer an integrative and quantitative approach to assessing the physiological status of organisms, linking external environmental stressors to internal biological responses. These biomarkers reveal how energy is distributed among essential functions (e.g., growth, reproduction, and maintenance), providing early warnings of stress before individual- or population-level effects become visible. In environmental risk assessment, this is particularly valuable at advanced stages, after emerging contaminants have already been identified as problematic through acute toxicity screenings. By detecting subtle, sub-individual-level effects, the CEA can guide the development of preventive measures to mitigate long-term ecological harm. Future research into these biomarkers is critical to improving our understanding of how contaminants affect aquatic ecosystems and shaping more effective environmental policies to protect ecosystem health.

## Figures and Tables

**Table 1:** Chemical properties of the antibiotics (sulfamethoxazole and trimethoprim) and aromatic amines (4-chloroaniline and 3,4-dichloroaniline). The literature data on the toxicity of the contaminants under study in *D. rerio* were used in the selection of treatments. Stock solutions and treatments (mg/L) for each compound are also present.

Compounds Name	Properties					Literature Data (mg/L)	Stock Solutions and Treatments (mg/L)
	CAS nº	Classification Group	Molecular Formula	Molecular Weight (g/mol)	Purity (%)		
**Sulfamethoxazole**	723-46-6	Antibiotic	C_10_H_11_N_3_O_3_S	253.28	≥98.0	0.013 to 5.00 ^a^	Stock solution: 2.50 0.156 to 2.50 (Dilution factor 2×)
**Trimethoprim**	738-70-5	Antibiotic	C_14_H_18_N_4_O_3_	290.30	≥98.5	10.0 ^b^	Stock solution: 400 25.0 to 400 (Dilution factor 2×)
**4-chloroaniline**	106-47-8	Aromatic amine	C_6_H_6_ClN	127.57	98.0	41.2 ^c^	Stock solution: 20.0 5.21 to 20.0 (Dilution factor 1.4×)
**3,4-dichloroaniline**	95-76-1	Aromatic amine	C_6_H_5_Cl_2_N	162.02	98.0	4.00 ^d^	Stock solution: 4.00 0.38 to 4.00 (Dilution factor 1.8×)

^a^ Range of concentrations revealing effects on survival, morphological malformations, and hatchability (Iftikhar et al., [28]); ^b^ NOEC value (Carlsson et al., [26]); ^c^ LC_50_ value (Heugens and Verbruggen, [25]); ^d^ LC_30_ value (OECD [27]).

## Data Availability

The original contributions presented in this study are included in this article. Further inquiries can be directed to the corresponding author.

## References

[1] Morin-Crini N., Lichtfouse E., Liu G., Balaram V., Ribeiro A.R.L., Lu Z., Stock F., Carmona E., Teixeira M.R., Picos-Corrales L.A. (2021). Emerging Contaminants: Analysis, Aquatic Compartments and Water Pollution. Emerging Contaminants Vol. 1: Occurrence and Impact.

[2] Wang F., Xiang L., Sze-Yin Leung K., Elsner M., Zhang Y., Guo Y., Pan B., Sun H., An T., Ying G. (2024). Emerging Contaminants: A One Health Perspective. Innovation.

[3] Rebelo D., Antunes S.C., Rodrigues S. (2023). The Silent Threat: Exploring the Ecological and Ecotoxicological Impacts of Chlorinated Aniline Derivatives and the Metabolites on the Aquatic Ecosystem. J. Xenobiotics.

[4] Diogo B.S., Rodrigues S., Antunes S.C. (2023). Antibióticos. Do Passado Ao Presente, Passando Pelo Ambiente. Rev. Ciência Elem..

[5] De Coen W.M., Janssen C.R. (2003). The Missing Biomarker Link: Relationships between Effects on the Cellular Energy Allocation Biomarker of Toxicant-Stressed *Daphnia magna* and Corresponding Population Characteristics. Environ. Toxicol. Chem..

[6] Cortes L., Marinov D., Sanseverino I., Cuenca A., Niegowska M., Rodriguez E., Lettieri T. (2020). Selection of Substances for the 3rd Watch List under the Water Framework Directive.

[7] Cortes L.G., Marinov D., Sanseverino I., Cuenca A.N., Niegowska M., Rodriguez E.P., Lettieri T. (2022). Selection of Substances for the 4th Watch List Under the Water Framework Directive.

[8] Diogo B.S., Rodrigues S., Golovko O., Antunes S.C. (2024). From Bacteria to Fish: Ecotoxicological Insights into Sulfamethoxazole and Trimethoprim. Environ. Sci. Pollut. Res..

[9] Duan W., Cui H., Jia X., Huang X. (2022). Occurrence and Ecotoxicity of Sulfonamides in the Aquatic Environment: A Review. Sci. Total Environ..

[10] Carvalho I., Santos L. (2016). Antibiotics in the Aquatic Environments: A Review of the European Scenario. Environ. Int..

[11] Sanpradit P., Byeon E., Lee J.-S., Jeong H., Kim H.S., Peerakietkhajorn S., Lee J.-S. (2024). Combined Effects of Nanoplastics and Elevated Temperature in the Freshwater Water Flea *Daphnia magna*. J. Hazard. Mater..

[12] Rodrigues S., Teixeira M.I., Diogo B.S., Antunes S.C. (2023). Assessment of the Ecotoxicological Effects of Deltamethrin to *Daphnia magna*: Linking Sub-Individual and Supra-Individual Parameters. Watershed Ecol. Environ..

[13] De Coen W.M., Janseen C.R. (1997). The Use of Biomarkers in *Daphnia magna* Toxicity Testing. IV. Cellular Energy Allocation: A New Methodology to Assess the Energy Budget of Toxicant-Stressed Daphnia Populations. J. Aquat. Ecossystem Stress Recovery.

[14] Smolders R., Bervoets L., De Coen W., Blust R. (2004). Cellular Energy Allocation in Zebra Mussels Exposed along a Pollution Gradient: Linking Cellular Effects to Higher Levels of Biological Organization. Environ. Pollut..

[15] Lam P.K.S. (2009). Use of Biomarkers in Environmental Monitoring. Ocean Coast. Manag..

[16] Abe F.R., Soares A.M.V.M., Oliveira D.P.d., Gravato C. (2018). Toxicity of Dyes to Zebrafish at the Biochemical Level: Cellular Energy Allocation and Neurotoxicity. Environ. Pollut..

[17] De Coen W.M., Wim M., Janssen C., Persoone G. (1995). Biochemical Assessment of Cellular Energy Allocation in Daphnia magna Exposed to Toxic Stress as an Alternative to the Conventional ‘Scope for Growth’ Methodology. Proceedings of the International Symposium on Biological Markers of Pollution.

[18] Gravato C., Almeida J.R., Silva C., Oliveira C., Soares A.M.V.M. (2014). Using a Multibiomarker Approach and Behavioural Responses to Assess the Effects of Anthracene in *Palaemon serratus*. Aquat. Toxicol..

[19] Rodrigues A.C.M., Gravato C., Quintaneiro C., Golovko O., Žlábek V., Barata C., Soares A.M.V.M., Pestana J.L.T. (2015). Life History and Biochemical Effects of Chlorantraniliprole on *Chironomus riparius*. Sci. Total Environ..

[20] Sokolova I.M., Frederich M., Bagwe R., Lannig G., Sukhotin A.A. (2012). Energy Homeostasis as an Integrative Tool for Assessing Limits of Environmental Stress Tolerance in Aquatic Invertebrates. Mar. Environ. Res..

[21] Aderemi A.O., Novais S.C., Lemos M.F.L., Alves L.M., Hunter C., Pahl O. (2018). Oxidative Stress Responses and Cellular Energy Allocation Changes in Microalgae Following Exposure to Widely Used Human Antibiotics. Aquat. Toxicol..

[22] Carvalho R., Ceriani L., Ippolito A., Lettieri T. (2015). Development of the First Watch List under the Environmental Quality Standards Directive.

[23] Tourinho P.S., Silva A.R.R., Santos C.S.A., Prodana M., Ferreira V., Habibullah G., Kočí V., van Gestel C.A.M., Loureiro S. (2022). Microplastic Fibers Increase Sublethal Effects of AgNP and AgNO in *Daphnia magna* by Changing Cellular Energy Allocation. Environ. Toxicol. Chem..

[24] Saraiva A.S., Sarmento R.A., Gravato C., Rodrigues A.C.M., Campos D., Simão F.C.P., Soares A.M.V.M. (2020). Strategies of Cellular Energy Allocation to Cope with Paraquat-Induced Oxidative Stress: Chironomids vs Planarians and the Importance of Using Different Species. Sci. Total Environ..

[25] Heugens E.H.W., Verbruggen E.M.J. (2009). Environmental Risk Limits for Monochloroanilines.

[26] Carlsson G., Patring J., Kreuger J., Norrgren L., Oskarsson A. (2013). Toxicity of 15 Veterinary Pharmaceuticals in Zebrafish (*Danio rerio*) Embryos. Aquat. Toxicol..

[27] (2013). OECD Test, No. 236: Fish Embryo Acute Toxicity (FET) Test.

[28] Iftikhar N., Konig I., English C., Ivantsova E., Souders C.L., Hashmi I., Martyniuk C.J. (2023). Sulfamethoxazole (SMX) Alters Immune and Apoptotic Endpoints in Developing Zebrafish (*Danio rerio*). Toxics.

[29] Rebelo D., Correia A.T., Nunes B. (2021). Acute and Chronic Effects of Environmental Realistic Concentrations of Simvastatin in *Danio rerio*: Evidences of Oxidative Alterations and Endocrine Disruptive Activity. Environ. Toxicol. Pharmacol..

[30] Mirth C.K., Anthony Frankino W., Shingleton A.W. (2016). Allometry and Size Control: What Can Studies of Body Size Regulation Teach Us about the Evolution of Morphological Scaling Relationships?. Curr. Opin. Insect Sci..

[31] Lucas J., Schouman A., Lyphout L., Cousin X., Lefrancois C. (2014). Allometric Relationship between Body Mass and Aerobic Metabolism in Zebrafish *Danio rerio*. J. Fish Biol..

[32] Pereira J.L., Marques C.R., Gonçalves F. (2004). Allometric Relations for *Ceriodaphnia* spp. and *Daphnia* spp.. Ann. Limnol.—Int. J. Limnol..

[33] Diogo B.S., Antunes S.C., Pinto I., Amorim J., Teixeira C., Teles L.O., Golovko O., Žlábek V., Carvalho A.P., Rodrigues S. (2023). Insights into Environmental Caffeine Contamination in Ecotoxicological Biomarkers and Potential Health Effects of *Danio rerio*. Heliyon.

[34] Ellman G.L., Courtney K.D., Andres V., Featherstone R.M. (1961). A New and Rapid Colorimetric Determination of Acetylcholinesterase Activity. Biochem. Pharmacol..

[35] Bradford M.M. (1976). A Rapid and Sensitive Method for the Quantitation of Microgram Quantities of Protein Utilizing the Principle of Protein-Dye Binding. Anal. Biochem..

[36] Novais S.C., Soares A.M.V.M., De Coen W., Amorim M.J.B. (2013). Exposure of *Enchytraeus albidus* to Cd and Zn—Changes in Cellular Energy Allocation (CEA) and Linkage to Transcriptional, Enzymatic and Reproductive Effects. Chemosphere.

[37] Ezeonyejiaku C.D., Ifedigbo I.I., Okoye C.O., Ezenwelu C.O. (2019). Cellular Energy Budget in Tropical Freshwater Fish Following Exposure to Sublethal Concentrations of Cadmium. J. Toxicol. Environ. Health Sci..

[38] Smolders R., De Boeck G., Blust R. (2003). Changes in Cellular Energy Budget as a Measure of Whole Effluent Toxicity in Zebrafish (*Danio rerio*). Environ. Toxicol. Chem..

[39] Vandenbrouck T., Soetaert A., van der Ven K., Blust R., De Coen W. (2009). Nickel and Binary Metal Mixture Responses in *Daphnia magna*: Molecular Fingerprints and (Sub)Organismal Effects. Aquat. Toxicol..

[40] Kanungo S., Wells K., Tribett T., El-Gharbawy A. (2018). Glycogen Metabolism and Glycogen Storage Disorders. Ann Transl Med.

[41] Cherkas A., Holota S., Mdzinarashvili T., Gabbianelli R., Zarkovic N. (2020). Glucose as a Major Antioxidant: When, What for and Why It Fails?. Antioxidants.

[42] Chiang J. (2014). Liver Physiology: Metabolism and Detoxification. Pathobiology of Human Disease.

[43] Xu M., Legradi J., Leonards P. (2022). Using Comprehensive Lipid Profiling to Study Effects of PFHxS during Different Stages of Early Zebrafish Development. Sci. Total Environ..

[44] Lei Y., Li F., Mortimer M., Li Z., Peng B.-X., Li M., Guo L.-H., Zhuang G. (2023). Antibiotics Disrupt Lipid Metabolism in Zebrafish (*Danio rerio*) Larvae and 3T3-L1 Preadipocytes. Sci. Total Environ..

[45] Park J.-S., Song J., Park J.-S., Lee S., Lee J., Park H.-J., Kim W.-K., Yoon S., Chun H.-S. (2020). 3,4-Dichloroaniline Promotes Fatty Liver in Zebrafish Larvae. Mol. Cell. Toxicol..

[46] Burkhardt-Holm P., Oulmi Y., Schroeder A., Storch V., Braunbeck T. (1999). Toxicity of 4-Chloroaniline in Early Life Stages of Zebrafish (*Danio rerio*): II. Cytopathology and Regeneration of Liver and Gills after Prolonged Exposure to Waterborne 4-Chloroaniline. Arch. Environ. Contam. Toxicol..

[47] Oulmi Y., Braunbeck T. (1996). Toxicity of 4-Chloroaniline in Early Life-Stages of Zebrafish (*Brachydanio rerio*): I. Cytopathology of Liver and Kidney after Microinjection. Arch. Environ. Contam. Toxicol..

[48] Van der Oost R., Beyer J., Vermeulen N.P. (2003). Fish Bioaccumulation and Biomarkers in Environmental Risk Assessment: A Review. Environ. Toxicol. Pharmacol..

[49] Shang Y., Wang X., Chang X., Sokolova I.M., Wei S., Liu W., Fang J.K.H., Hu M., Huang W., Wang Y. (2021). The Effect of Microplastics on the Bioenergetics of the Mussel *Mytilus coruscus* Assessed by Cellular Energy Allocation Approach. Front. Mar. Sci..

[50] Shenton D., Smirnova J.B., Selley J.N., Carroll K., Hubbard S.J., Pavitt G.D., Ashe M.P., Grant C.M. (2006). Global Translational Responses to Oxidative Stress Impact upon Multiple Levels of Protein Synthesis. J. Biol. Chem..

[51] Dasuri K., Zhang L., Keller J.N. (2013). Oxidative Stress, Neurodegeneration, and the Balance of Protein Degradation and Protein Synthesis. Free Radic. Biol. Med..

[52] van Dam L., Dansen T.B. (2020). Cross-Talk between Redox Signalling and Protein Aggregation. Biochem. Soc. Trans..

[53] Singh P., Kesharwani R.K., Keservani R.K. (2017). Protein, Carbohydrates, and Fats: Energy Metabolism. Sustained Energy for Enhanced Human Functions and Activity.

[54] Moolman L., Van Vuren J.H.J., Wepener V. (2007). Comparative Studies on the Uptake and Effects of Cadmium and Zinc on the Cellular Energy Allocation of Two Freshwater Gastropods. Ecotoxicol. Environ. Saf..

[55] Rodrigues S., Antunes S.C., Correia A.T., Nunes B. (2019). Toxicity of Erythromycin to *Oncorhynchus mykiss* at Different Biochemical Levels: Detoxification Metabolism, Energetic Balance, and Neurological Impairment. Environ. Sci. Pollut. Res..

[56] Kovačević M., Stjepanović N., Hackenberger D.K., Lončarić Ž., Hackenberger B.K. (2022). Comprehensive Study of the Effects of Strobilurin-Based Fungicide Formulations on *Enchytraeus albidus*. Ecotoxicology.

[57] Gomes S.I.L., Soares A.M.V.M., Amorim M.J.B. (2015). Changes in Cellular Energy Allocation in *Enchytraeus crypticus* Exposed to Copper and Silver—Linkage to Effects at Higher Level (Reproduction). Environ. Sci. Pollut. Res..

[58] Simcic T., Lukancic S., Brancelj A. (2005). Comparative Study of Electron Transport System Activity and Oxygen Consumption of Amphipods from Caves and Surface Habitats. Freshw. Biol..

[59] Båmstedt U. (1980). ETS Activity as an Estimator of Respiratory Rate of Zooplankton Populations. The Significance of Variations in Environmental Factors. J. Exp. Mar. Biol. Ecol..

[60] Erk M., Ivanković D., Strižak Ž. (2011). Cellular Energy Allocation in Mussels (*Mytilus galloprovincialis*) from the Stratified Estuary as a Physiological Biomarker. Mar. Pollut. Bull..

[61] Alzahrani H. (2022). Assessing the Sub-Lethal Effects of Copper, Cadmium, Pentachlorophenol and 3,4-Dichloroaniline on Freshwater Rotifers *Brachionus calyciflorus* Using the Energy Budget Biomarkers. Ph.D. Thesis.

[62] Rebelo D., Antunes S.C., Rodrigues S. (2025). Hidden Dangers: Aromatic Amines and Their Impact on Freshwater Species. Chem. Ecol..

